# Understanding Drivers of Ocular Fibrosis: Current and Future Therapeutic Perspectives

**DOI:** 10.3390/ijms222111748

**Published:** 2021-10-29

**Authors:** Fabiana Mallone, Roberta Costi, Marco Marenco, Rocco Plateroti, Antonio Minni, Giuseppe Attanasio, Marco Artico, Alessandro Lambiase

**Affiliations:** 1 Department of Organ of Sense, Sapienza University of Rome, 00161 Rome, Italy; fabiana.mallone@uniroma1.it (F.M.); rocco.plateroti@uniroma1.it (R.P.); antonio.minni@uniroma1.it (A.M.); giuseppe.attanasio@uniroma1.it (G.A.); marco.artico@uniroma1.it (M.A.); alessandro.lambiase@uniroma1.it (A.L.); 2 Department of Drug Chemistry and Technology, Istituto Pasteur-Fondazione Cenci Bolognetti, Sapienza University of Rome, 00161 Rome, Italy; roberta.costi@uniroma1.it

**Keywords:** ocular fibrosis, ocular inflammation, age-related macular degeneration, diabetic retinopathy, glaucoma, optic neuropathy, TGFβ/Smad pathway, gene therapy, chemical inhibitors, angiogenesis, biomechanics, cell-based therapy

## Abstract

Ocular fibrosis leads to severe visual impairment and blindness worldwide, being a major area of unmet need in ophthalmology and medicine. To date, the only available treatments are antimetabolite drugs that have significant potentially blinding side effects, such as tissue damage and infection. There is thus an urgent need to identify novel targets to prevent/treat scarring and postsurgical fibrosis in the eye. In this review, the latest progress in biological mechanisms underlying ocular fibrosis are discussed. We also summarize the current knowledge on preclinical studies based on viral and non-viral gene therapy, as well as chemical inhibitors, for targeting TGFβ or downstream effectors in fibrotic disorders of the eye. Moreover, the role of angiogenetic and biomechanical factors in ocular fibrosis is discussed, focusing on related preclinical treatment approaches. Moreover, we describe available evidence on clinical studies investigating the use of therapies targeting TGFβ-dependent pathways, angiogenetic factors, and biomechanical factors, alone or in combination with other strategies, in ocular tissue fibrosis. Finally, the recent progress in cell-based therapies for treating fibrotic eye disorders is discussed. The increasing knowledge of these disorders in the eye and the promising results from testing of novel targeted therapies could offer viable perspectives for translation into clinical use.

## 1. Introduction

Ocular fibrosis is a complex biological process responsible for the pathogenesis or treatment failure of many blinding eye diseases, including corneal and conjunctival scarring, open-angle glaucoma and failure of glaucoma filtration surgery (GFS), fibrosis in the lens capsule post-cataract surgery, scarring in the tissue around the extraocular muscles in the strabismus surgery, subretinal fibrosis in neovascular age-related macular degeneration (nAMD), fibrovascular proliferative tissue in diabetic retinopathy, and failure of retinal detachment surgery due to proliferative vitreoretinopathy (PVR) [1,2].

In these conditions, the main components of the disease process include inflammation, fibroblast activation and extracellular matrix (ECM) accumulation, and resultant tissue contraction. Moreover, deficiency in the limbal stem cells is involved as it leads to the formation of vascularized scar tissue of conjunctival origin on the corneal surface [1].

Advances in our understanding of the biological mechanisms underlying ocular fibrosis is leading to the introduction of new therapeutic agents targeting a wide range of key processes.

This review provides current and futures perspectives on different approaches for treating fibrotic eye disorders and focuses on recent progress in the area of gene and cell-based therapies.

## 2. The Fibrogenic Process

The nature of fibrotic disease in the eye is quite similar to that seen in fibrotic disorders in other tissues of the human body. However, in the eye, fibrosis can have disastrous consequences for vision, mechanically disrupting the visual axis [1] or sufficiently disturbing the tissue microenvironment such that proper cellular functioning is no longer possible.

Similar to tissues elsewhere in the human body, homeostasis in the eye is based on the presence normal vasculature, organized extracellular matrix (ECM) structure, and various cell types. If homeostasis is disturbed by infections, inflammations, or metabolic dysfunctions, a wound healing response is activated that involves alteration of vascular permeability, infiltration of inflammatory cells, proliferation and activation of fibroblasts, ECM accumulation, and tissue contraction [3,4,5].

ECM synthesis, in the process of tissue repair, can result from activated fibroblasts converting to myofibroblasts or from epithelial cells undergoing epithelium–mesenchymal transition (EMT). ECM synthesis is balanced by ECM degradation from matrix metalloproteinases (MMPs) for maintenance of normal tissue architecture.

**Figure 1:**
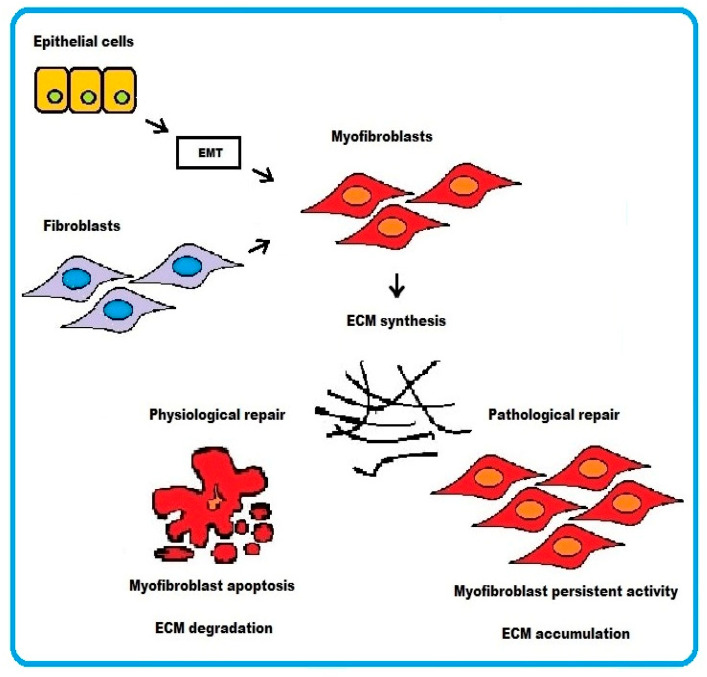
Schematic representation of the cellular mechanisms underlying fibrosis. Two pathways are depicted: (i) epithelial cells transforming into myofibroblasts via EMT or (ii) activated fibroblasts differentiating into myofibroblasts. In physiological wound healing, myofibroblasts disappear by apoptosis. Conversely, the persistence of myofibroblasts activity in pathological wound healing process results in excessive accumulation of ECM.

In physiological wound healing, myofibroblasts disappear by apoptosis after restoring tissue integrity. However, severe or repetitive tissue injury can result in persistence of myofibroblast activity leading to excessive accumulation and contraction of disorganized ECM. The end result is formation of irreversible fibrotic scar, which ultimately represents some sort of resolution of the damaged tissue (Figure 1).

## 3. Fibrotic Disorders in the Eye

Fibrosis of the cornea can occur following infectious, traumatic, metabolic, or immune-mediated injury involving the epithelium and underlying stroma and/or the endothelium and posterior stroma. The resultant disturbance of stromal transparency thereby leads to loss of vision [6,7].

**Figure 2:**
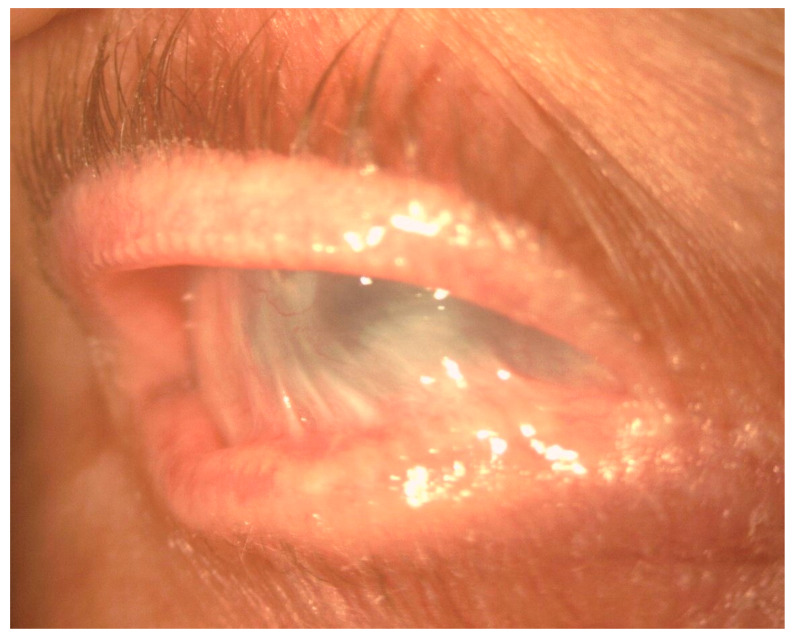
Slit-lamp image showing severe conjunctival fibrosis with symblepharon and corneal scarring in OMMP.

**Figure 3:**
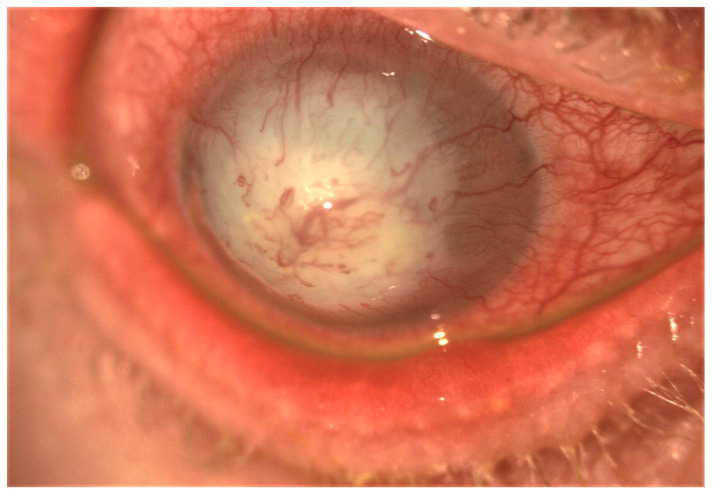
Slit-lamp image showing post-infectious severe corneal scarring and neovascularization.

In the conjunctiva, causes of cicatrization include immune-mediated disorders such as ocular mucous membrane pemphigoid (OMMP), thermal and chemical burns, post-infectious conjunctivitis, Stevens–Johnson syndrome (SJS), and toxic epidermal necrolysis (Lyell syndrome). Common to these disorders are the development of cicatricial entropion and trichiasis, fornix foreshortening, symblepharon, ocular dryness, and limbal stem cell deficiency (Figure 2). These changes can lead to formation of vascularized scar tissue of conjunctival origin on the corneal surface, corneal ulceration, super-infection, and perforation [8] (Figure 3).

In addition, tissue fibrosis at the level of trabecular meshwork (TM), lamina cribrosa (LC), and Schlemm canal (SC) cells has been identified as prerequisite for glaucoma onset and progression, resulting in reduced outflow of aqueous humor and increased intraocular pressure (IOP) [9]. 

Moreover, excess fibroblast proliferation and subconjunctival fibrosis is reported in filtering bleb failure after GFS [10]. Topical mitomycin C (MMC) is applied to local tissue after trabeculectomy to prevent excess proliferation of subconjunctival fibroblasts. 

Excess matrix synthesis by lens epithelial cells (LECs) is responsible for opacification of the anterior lens capsule in response to eye trauma or uveitis, as well as opacification and wrinkle of the posterior capsule secondary to residual LECs after cataract surgery [11].

In the orbit, fibrotic tissue remodeling is involved in the spectrum of dysthyroid orbitopathies, whereas excess scarring in the tissues adjacent to the extraocular muscles can affect the outcome of strabismus surgery.

**Figure 4:**
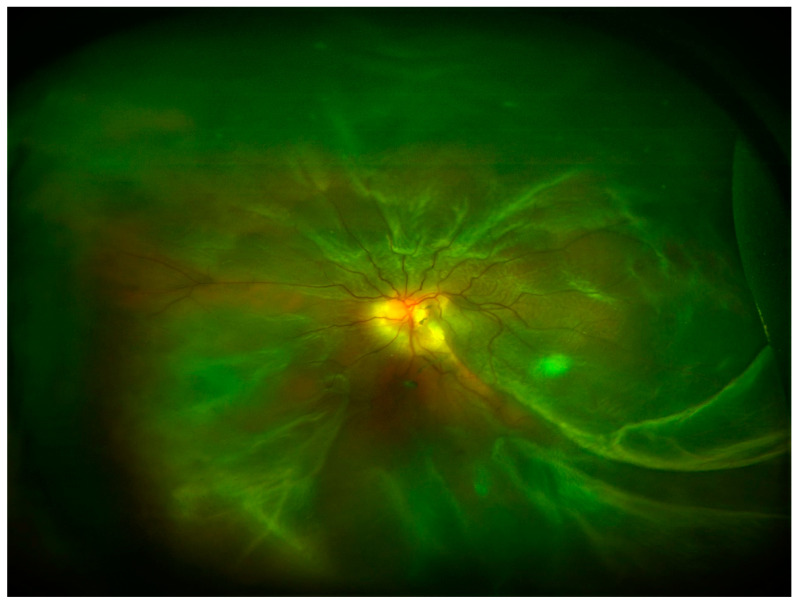
Ultra-wide field retinography image showing retinal detachment with PVR.

In the posterior segment of the eye, uncontrolled retinal vascular proliferation, as a result of diabetes-associated retinal hypoxia, can lead to fibrosis and tractional retinal detachment [12]. Under the retina, similar fibrosis can result subsequent to subretinal hemorrhage associated with nAMD [13]. Moreover, fibrosis can occur following retinal detachment or previous vitreoretinal surgery leading to PVR and tractional retinal detachment [14] (Figure 4). In all the cases, excessive deposition of ECM and appearance of myofibroblasts and inflammatory cells are reported in the eye.

Inflammatory/fibrogenic/angiogenetic growth factors/cytokines produced by injured tissues in the eye, as well as biomechanical factors, play a pivotal role in fibrotic tissue formation.

## 4. Molecular and Cellular Mechanisms Underlying Ocular Fibrosis: Upstream and Downstream Regulators

### 4.1. Growth Factors—TGFβ

The ocular fibrogenic process, either derived from fibroblast–myofibroblast conversion (i.e., subconjunctival fibroblasts, keratocytes, or bone marrow-derived cells) or from EMT (i.e., lens or retinal pigment epithelium), is strictly regulated by growth factors including the transforming growth factorβ (TGFβ) [15,16].

TGFβ is the most important ligand involved in the regulation of cell proliferation and differentiation, ECM production, angiogenesis, and immune modulation in ocular tissues in physiological or pathological processes of development or tissue repair [17,18]. Aberrant TGFβ signaling has been associated with unfavorable inflammatory responses and fibrotic disease in the eye [18]. 

Three isoforms of TGFβ, namely, β1, β2, and β3, are known in mammals. TGFβ isoforms play distinct functions in wound healing, with TGF-β1/2 having predominantly pro-scarring roles and TGFβ3 having mainly anti-scarring effects [19,20,21]. All TGFβ isoforms and receptors are present in ocular tissues [17].

All TGFβ1-β3 isoforms utilize the small mothers against decapentaplegic (Smad) signaling pathway, which is specific to the members of the TGFβ superfamily [17].

#### 4.1.1. The SMAD Pathway

The Smad family includes three different functional classes: receptor-activated Smads (R-Smads), common mediator Smads (Co-Smads), and inhibitory Smads (I-Smads). Smad2 and Smad3 are the R-Smads phosphorylated upon TGFβ binding to receptor type I kinase [22]. Smads2/3 then partner with the Co-Smad4 and translocate to the nucleus, where they modulate the expression of TGFβ-responsive genes. Smads6/7 are known to be I-Smads that block phosphorylation of Smads2/3 [23].

The expression of the majority of the ECM components involved in matrix structure reorganization depends on Smad3, whereas expression of MMPs is Smad2-dependent [24,25,26]. Accordingly, lack of Smad3 has been associated to reduced secretion of ECM components including αSMA, fibronectin, and collagen type I in human LECs [27]. Furthermore, mice null for Smad3 exhibited lower expression of ECM components in corneal stromal cells, LECs, and in RPE cells following injury, compared to WT mice [28,29,30,31]. On the other hand, the TGFβ/Smad2 axis upregulated the expression of MMP-2 and MMP-13 in corneal epithelial cells [24].

Other growth factors/cytokines are known to further modulate the TGFβ/Smad activity in ocular tissues. Tumor necrosis factor α (TNFα) counteracts the activities of TGFβ/Smad in the process of wound healing. Loss of TNFα in a model of healing-corneal alkali burn in mice resulted in increased inflammation, fibrosis, and neovascularization promoted by TGFβ [32]. In addition, TNFα inhibited the induction effects of both TGFβ1 and VEGF on cultured vascular endothelial cells and blocked corneal stromal neovascularization in mice [33]. Moreover, interferon-γ-activated STAT signal was reported to hinder the TGFβ/Smad signal by up-regulating Smad7 in cultured human subconjunctival fibroblasts [34,35]. The connective tissue growth factor (CTGF), a fibrogenic cytokine, is a downstream mediator of TGFβ/Smad-induced fibrosis. CTGF was found to be overexpressed in the filtration blebs after trabeculectomy [36]. Moreover, CTGF activity was associated with the process of EMT and ECM synthesis by human RPE cell line ARPE19 in vitro [37]. A possible cross-talk is also proposed between the nerve growth factor (NGF) and TGFβ. NGF modulates TGFβ activity by promoting survival/apoptosis of TGFβ1-activated myofibroblasts on the basis of surface trkA^NGFR^/p75^NTR^ rate expression in vitro experiments [38]. Interestingly, NGF was reported to affect αSMA/p75^NTR^ expression and TGFβ1 release in conjunctival fibroblasts from human OMMP [39].

#### 4.1.2. Non-Smad Pathways

While the Smad pathway represents the canonical signaling pathway for TGFβ, several non-Smad intracellular signaling cascades have been implicated in mediating the cellular effects of TGFβ in ocular fibrosis.

TGFβ also signals through the mitogen activated protein kinase (MAPK) pathways [40]. The TGFβ-signaling cascades via MAPK include three subfamilies, the extracellular signal-regulated kinases (ERKs), the c-Jun N-terminal kinases (JNKs), and the p38 mitogen-activated protein kinases (p38s) [41,42]. In general, the ERKs are activated by growth factors, while cellular stresses and inflammatory cytokines rather activate JNKs and p38s. These signaling pathways mediate different biological responses in the fibrotic process in the eye. For example, activation of ERKs mediated the TGFβ1-induced EMT and fibrosis in ARPE-19 cells [42]. Moreover, the JNKs were identified to promote in vivo corneal epithelial migration following injury [43]. Similarly, the p38s were demonstrated to serve a key role in corneal endothelial wound healing, being involved in human corneal endothelial cell migration induced by TGFβ2 [44]. Additionally, p38 pathway was found to mediate the expression of type I collagen induced by TGFβ2 in ARPE-19 cells [45].

The RhoA/Rho-kinase pathway is another non-Smad intracellular signaling pathway for TGFβ involved in ocular fibrosis. RhoA downstream main effectors are the Ras-related C3 botulinum toxin substrate 1 (Rac1) and the Rho-associated, coiled-coil-containing kinases (ROCK), which play a critical role in the regulation of cellular actomyosin cytoskeletal organization and motility. The RhoA/Rho-kinase pathway was shown to mediate the expression of type I collagen induced by TGFβ2 in human RPE cells ARPE-19 [46]. The myocardin-related transcription factor-A/serum response factor (MRTF-A/SRF) pathway is activated in response to RhoA activation. This pathway was demonstrated to regulate TGFβ-induced EMT, as well as αSMA expression, in LECs [47,48].

There are various forms of cross-talks between Smad and non-Smad signaling pathways. Specifically, the middle linker region of Smad2 or Smad3 can be phosphorylated by MAPKs and RhoA in response to various ligands. There are in vivo and in vitro evidences suggesting that phosphorylation in the Smad middle liker region by such signals further promoted or suppressed Smad-dependent gene expression [49,50,51]. In addition, the phosphatidylinositol-3-kinase (PI3K)/Akt pathway was identified to mediate the expression of type I collagen induced by TGFβ2 in ARPE-19 cell line through Smad-dependent and Smad-independent pathways [52]. These results suggest a crosstalk also between PI3K/Akt, the Smad, and the non Smad MAPK and RhoA/Rho-kinase pathways in fibrotic disorders to the eye.

**Figure 5:**
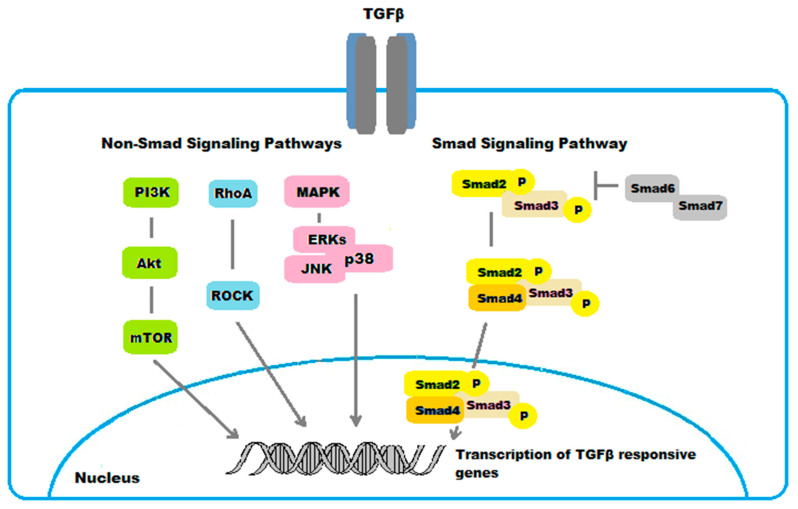
Schematic representation of TGFβ/Smad- and non-Smad-regulated pathways in ocular fibrogenic disorders.

The main TGFβ/Smad- and non-Smad-regulated pathways involved in ocular fibrogenic disorders are shown in Figure 5.

## 5. Preclinical Studies with Drugs Affecting TGFβ for Prevention/Treatment of Ocular Fibrosis

TGFβ/Smad signaling is the most investigated target to be inhibited in the prevention/treatment of inflammatory/fibrotic diseases in the eye. Smad7, which effectively blocks Smads2/3 signaling, exhibits promising therapeutic effect in ocular fibrotic disease in experimental animals. Specifically viral and non-viral gene transfer techniques based on TGFβ/Smads2/3/7 have been studied in vitro and in vivo animal models. Moreover, non-gene transfer techniques based on TGFβ/Smads2/3/7 have been investigated in preclinical studies.

### 5.1. Anti-TGFβ/Smad Viral and Non-Viral Gene Transfer Treatment Strategies for Prevention/Treatment of Ocular Fibrosis

It has been reported that blocking the activity of TGFβ by systemic expression of soluble type II TGFβ receptor by adenoviral gene transfer resulted in suppressed scarring and neovascularization in healing after alkali burn rat cornea [53]. In addition, small interference RNAs (siRNAs) transfection of type II TGFβ receptor into cultured human corneal fibroblasts and in eyes of a mouse model of ocular inflammation and fibrosis effectively suppressed the fibrogenic/inflammatory reaction in vitro and in vivo [54]. However, therapies that target TGFβ expression/activation or binding of TGFβ to its receptor may potentially perturb healing of the epithelial component by interfering with the p38MAPK activity, which is critical for migration of epithelial cells during tissue repair [55,56]. In addition, such strategies may induce a number of unwanted side effects due to the pleiotropic biological actions of TGFβ mediated by multiple signaling pathways.

Compounds that selectively target signaling pathways downstream of the TGFβ receptor are expected to achieve the desired effects while avoiding complications. Specifically, the blockage of TGFβ activity at the level of Smad signaling may obtain a more favorable result since the other TGFβ signaling cascades would remain intact.

Using a mouse corneal alkali burn model, knocking out of Smad3 by gene targeting suppressed tissue fibrosis of the healing cornea in association with a reduction of macrophage infiltration, myofibroblast generation, and growth factor expression [2,57]. In accordance, adenoviral gene transfer of Smad7 cDNA by topical application suppressed scarring and neovascularization in a healing mouse cornea after alkali exposure in association with significant decrease in expression of profibrogenic and proinflammatory cytokines [58]. The effects were more marked than those seen in Smad3-null mice, probably because Smad7 also suppresses the nuclear factor-κB (NF-κB) pathway, which leads to suppression of inflammation cascades [58]. Specifically, Smad7 does block nuclear translocation of NF-κB, a signal transmitter related to inflammation [17]. Moreover, transfer of Smad7 by adenoviral vector suppressed TGFβ1-mediated up-regulation of fibrogenic and inflammatory components in cultured human subconjunctival fibroblasts [59]. In addition, Smad7 gene transfer reduced the fibrogenic reaction in healing, incision-injured mouse conjunctiva, suggesting a therapeutic potential in the prevention of excess scarring in the filtering bleb post-trabeculectomy [59]. To prevent fibrous capsular opacification, researchers investigated Smad7 gene transfer by adenoviral vector to an injured lens epithelium in mice. This resulted in suppressed injury-induced lens epithelium EMT [60]. These favorable results indicate that strategies that promote Smad7 activity might have a therapeutic potential to prevent secondary cataracts. In addition, inhibition of the TGFβ/Smad signaling was investigated for the prevention and treatment of PVR. Specifically, it was demonstrated that the Smad7 gene transfer inhibited fibrogenic responses to TGFβ2 by RPE cells in vitro and in vivo [61]. In detail, gene introduction of Smad7 in ARPE-19 human RPE cells inhibited the TGF-β2/Smad signaling and expression of collagen type I and TGFβ1. Moreover, Smad7 overexpression suppressed EMT and fibrogenic responses in RPE cells after retinal detachment in mice [61]. 

### 5.2. Other Anti-TGFβ/Smad Viral and Non-Viral Gene Transfer Treatment Strategies for Prevention/Treatment of Ocular Fibrosis

The inhibition of pathways modulating the TGFβ/Smad signaling has also been investigated in inflammatory/fibrotic diseases in the eye. 

The bone morphogenic protein-7 (BMP-7) is a member of the TGFβ superfamily, which has antagonistic effects on TGFβ/Smad signal in tissue fibrosis. Specifically, BMP-7 facilitates the expression of the inhibitors of differentiation 2 and 3 (Id2 and Id3), both of which block Smads2/3 phosphorylation. Gene introduction of BMP-7 has shown a therapeutic effect on corneal alkali burn model in mice, although its efficacy is less than that of Smad7 [62]. In another report, adenoviral gene transfer of Id2, Id3, or BMP-7 suppressed injury-induced EMT in mouse LECs in vivo [63]. Moreover, BMP-7 gene therapy to treat preformed corneal fibrosis using established rabbit in vivo and human in vitro models significantly restored transparency of the cornea [64]. 

In addition, the peroxisome proliferator-activated receptor (PPAR) family has a role in attenuating the TGFβ/Smad signal in fibrotic eye diseases. The PPAR family consists of three isoforms, namely, PPARα, β/δ, and γ, which are involved in control of the inflammatory response, fibrogenic reaction, and cell proliferation during wound healing [65,66]. PPARγ gene transfer was shown to suppress activation of ocular fibroblasts and macrophages in vitro upon exposure to TGFβ/Smad signal [67]. In vivo experiments showed that PPARγ gene transfer suppressed monocytes/macrophages invasion and suppressed the generation of myofibroblasts, as well as cytokines/growth factors and MMP upregulation in an alkali-burned mouse cornea [67]. 

The family of small leucine-rich proteoglycans (SLRPs) is also involved in the ocular wound healing process. The SLRPs are natural inhibitors of TFGβ/Smad activity. Decorin is an SLRP implicated in regulating assembly of collagen fibrils and ECM structure in the eye [68]. Transfection of decorin through mammalian expression vector inhibited TGFβ-driven elevated expression of profibrogenic genes, as well as formation of myofibroblasts in human corneal fibroblasts [68]. The same authors later demonstrated that decorin gene transfer with adeno-associated virus serotype 5 (AAV5) in vivo rabbit model of corneal fibrosis significantly reduced corneal haze [69]. Moreover, decorin gene therapy delivered to the rabbit corneal stroma with AAV5 suppressed corneal neovascularization in vivo [70]. 

The role of NADPH oxidase 4 (Nox4), a facilitator of TGFβ-induced fibrotic responses in the eye, was assessed in Nox4 knockout (KO) mouse model of GFS, demonstrating decreased post-surgical scarring [71]. Selective target of Nox4 gene expression by an adenovirus carrying a Nox4 small interfering RNA (siRNA) (Ad-Nox4i) in human Tenon’s fibroblasts (HTFs) was observed to reduce TGFβ1-mediated proliferation in HTFs [71]. Activation of Smads2/3 by phosphorylation of MAPK at its middle linker region is an additional target of inhibition of TGFβ for prevention of ocular fibrosis. Adenoviral gene transfer of dominant-negative p38MAPK abolished the post-retinal detachment fibrotic reaction of the RPE in vivo in a mouse model of PVR [72], supporting its effectiveness in preventing/treating PVR.

### 5.3. Anti-TGFβ/Smad Treatment Strategies by Non-Gene Transfer Techniques

There is evidence suggesting there is potential efficacy of blocking Smad signaling also by non-gene transfer techniques. 

On the basis of Smad7 blockage of NF-κB activity, researchers investigated the use of NF-κB inhibitors for prevention of ocular fibrosis [73]. Specifically, it was confirmed that inhibiting NF-κB by a peptide inhibitor, SN50, produced a therapeutic effect on alkali-burned corneas in mice [73]. The mechanism of action of SN50 is based on inhibition of the inflammatory response and promotion of epithelial cells proliferation through activation of TNFα/JNK signal [73]. 

The aldehyde dehydrogenase (ALDH) superfamily, composed of enzymes that catalyze aldehyde oxidation, has been identified to be involved in several profibrotic pathways, including TGFβ, in order to control fibroblast activation and tissue fibrosis [74]. There are studies demonstrating ALDH1 to be upregulated in OMMP conjunctiva and cultured fibroblasts [75]. Treatment with ALDH inhibitors, including disulfiram, to human OMMP fibroblasts proved effective in restoring their functionality in vitro. Moreover, topical application of disulfiram decreased fibrosis in vivo mouse model of scarring allergic eye disease (AED), used as a surrogate for OMMP [75]. 

It has also been reported that components of herbal medicines exert anti-fibrogenic/inflammatory effects by disrupting the TGFβ/Smad signaling. Halofuginone, a derivative of febrifugine, which is an herbal extract originally isolated from the plant Dichroa febrifuga, reportedly blocked tissue fibrosis by upregulating the expression of Smad7 and downregulating the expression of TGFβ receptor type II from in vitro and in vivo studies [76]. The antifibrotic potential of halofuginone was investigated in human corneal fibroblasts in vitro. Specifically, halofuginone downregulated the protein expression of Smad3 and reduced the expression of the fibrotic markers α-SMA, fibronectin, and type I collagen in human corneal fibroblasts [77]. In addition, components of an herbal medicine, Inchin-Ko-Tou; genipin [78]; and emodin [79] demonstrated antifibrogenic inflammatory effects in vitro in cultured ocular cells including LEC line and human subconjunctival fibroblasts, and in vivo in the healing of an alkali-burned cornea in mice. Tetrandrine, the major component of an herbal medicine, Boui, also suppressed fibrogenic reaction of cultured subconjunctival fibroblasts via blocking Smads2/3 signal by upregulation of Smad7 [80]. In addition, pirfenidone, an anti-fibrotic drug reported to decrease TGFβ expression and enhance the protective role of PPARs, was investigated in human ocular fibroblasts in vitro, demonstrating decreased cell proliferation and matrix synthesis [81].

Moreover, molecules aimed at blocking CTGF were identified to play an important role in the prevention of ocular fibrosis related to the TGFβ/Smad axis. The group of Wang et al. demonstrated that subconjunctival injection of a CTGF antibody was able to maintain larger bleb areas and lower intraocular pressures in a rabbit model of GFS [82].

With reference to the RhoA/Rho-kinase pathway, the AMA0526, Rho kinase inhibitor was found to inhibit proliferation of HTFs and TGFβ1-induced fibroblast-to-myofibroblast differentiation. Moreover, the effects of AMA0526 were investigated on wound healing process in a rabbit model of GFS, demonstrating improved surgical outcome [83]. Similarly, the role of Y-27632, a specific inhibitor of the RhoA/ROCK pathway, was evaluated in HTF activities including proliferation, adhesion, contraction, migratory response, and myofibroblast transdifferentiation. Results showed that use of Y-27632 significantly reduced collagen gel contraction and α-SMA expression in HTFs. Y-27632 also increased HTF motility. Effects of Y-27632 on prevention of postoperative scar formation were also examined in a rabbit model of GFS, showing significantly improved surgical outcome compared with the vehicle [84]. A Rac1 inhibitor also efficiently abolished fibroblast-mediated matrix contraction and MMP-1 expression in conjunctival tissue [85]. Furthermore, the role of Y-27632 was investigated to prevent fibrosis in human RPE cells. Specifically, Y-27632 suppressed the expression of ECM components induced by CTGF or TGFβ in ARPE-19 cells in vitro, suggesting a potential approach for prevention of PVR [86].

Moreover, several chemical inhibitors of the PI3K/Akt signaling were investigated in vitro and in vivo for prevention of TGFβ-mediated scar formation in eye disorders. Specifically, sulforaphane, a molecule capable of modulating PI3K/Akt activity, inhibited proliferation, migration, and synthesis of the ECM in human conjunctival fibroblast [87]. Similarly, lithium chloride was shown to abolish myofibroblast transdifferentiation via PI3K/Akt signal in HTFs [88]. Moreover, 3-methyladenine, a selective inhibitor of PI3K, exerted antifibrotic effects on experimental subretinal fibrosis in mice [89].

The therapeutic efficacy of SB202190, a p38MAPK chemical inhibitor, was evaluated in a ARPE-19 human RPE cell line, demonstrating reduced TGFβ2-mediated migration and ECM production [72]. In accordance, pharmacologic inhibition of p38 with SB203580 was found to suppress TGFβ-induced myofibroblast transdifferentiation in HTFs after GFS [90].

Moreover, Notch signaling is involved in the regulation of ocular fibrosis through TGFβ1/Smad, and its inhibition may have therapeutic value in the prevention and treatment of retinal fibrosis [91]. Inhibition of the Notch signaling by the γ-secretase inhibitors including RO4929097, LY411575, and DAPT demonstrated prevention of retinal fibrosis in in vitro and in vivo experiments [92,93]. In detail, intravitreal injection of RO4929097 inhibited retinal glial (Müller) cell gliosis and limited overexpression of ECM proteins in a murine model of retinal fibrosis [92]. LY411575 significantly attenuated the EMT of RPE cells in vitro and inhibited mouse PVR formation in vivo [91]. Blockade of the Notch pathway with DAPT suppressed TGFβ2-induced EMT in human RPE cells [93].

The main preclinical studies with gene transfer and non-gene transfer techniques targeting TGFβ/Smad in Ocular Fibrosis are summarized in Table 1.

## 6. Clinical Trials with Drugs Affecting TGFβ/Smad for Prevention/Treatment of Ocular Fibrosis

Currently, treatment of fibrotic disorders to the eye is mainly based on administration of systemic steroids and immunosuppressive agents such as azathioprine, methotrexate, cyclophosphamide, and mycophenolate mofetil, as well as monoclonal antibodies. However, some patients have contraindications, fail to respond to these conventional therapies, or experience intolerable side effects. Moreover, patients may develop resistance to their use. In addition, fibrotic disorders to the eye ultimately require challenging surgical treatments, which often result in poor functional and anatomical outcomes. For these reasons, alternative treatment modalities are urgently needed.

Currently, only a few clinical trials are available that are based on agents targeting TGFβ/Smad for prevention and treatment of fibrotic disorders to the eye.

A large, controlled, multicenter, randomized phase III clinical trial evaluated the efficacy of subconjunctival CAT-152 (lerdelimumab), a monoclonal antibody to TGFβ2, in preventing the progression of bleb scarring in glaucomatous patients undergoing first-time trabeculectomy. However, there was no reported difference in surgical success between CAT-152 and placebo [94].

In addition, a multicenter, multidose, dose escalation phase I/II study is currently active to evaluate the safety, tolerability, and clinical activity of intravitreally injected RXI-109, a self-delivering RNAi (sd-rxRNA) compound that selectively targets CTGF to reduce progression of subretinal fibrosis in subjects with advanced nAMD [95].

## 7. Angiogenic Factors

The increase in local hypoxic conditions and impaired cellular responses to hypoxia are critical factors that contribute to a delay in wound healing. Angiogenesis serves a major role in delayed wound healing response.

Vascular endothelial growth factor (VEGF) is a key mediator of angiogenesis and stimulates both fibroblasts and endothelial cells in late tissue repair. Several preclinical and clinical studies have shown promising results of anti-VEGF therapies (bevacizumab, ranibizumab) in preventing corneal vascularization [96]. Cho et al. showed that knocking down of VEGF improved corneal graft survival by decreasing angiogenesis in a murine penetrating keratoplasty model [97].

On the other hand, the role of anti-VEGF therapies in reducing scarring and fibrosis after GFS remains controversial. A randomized clinical trial was performed to compare trabeculectomy with adjunctive intracameral bevacizumab versus intraoperative MMC. Results reported that intracameral bevacizumab was as effective in increasing trabeculectomy success as MMC; however, it was associated with an increased risk of filtering bleb leakage [98]. Moreover, the use of intracameral bevacizumab was investigated as an adjunct to MMC trabeculectomy in several trials. A prospective, randomized, placebo-controlled trial reported that administration of intracameral bevacizumab in MMC trabeculectomy significantly reduced the need for needling interventions and led to a higher success rate after surgery [99]. However, a recent prospective, randomized, controlled trial reported that adjuvant use of intracameral bevacizumab during MMC trabeculectomy is not justified as it does not improve surgical success rates [100]. Thus, further RCTs are needed to investigate the efficacy and safety of anti-VEGF therapies after GFS.

Interestingly, Bergen et al. showed that intracameral injection of a monoclonal antibody to placental growth factor (PlGF), a VEGF-homolog that binds to VEGF-R1, resulted in increased bleb area and survival in a mouse model of glaucoma surgery [101]. Furthermore, anti-PlGF treatment was reported to enhance the efficacy of VEGF inhibitors, suggesting possible combinatory strategies [102].

A number of intravitreal anti-VEGF agents are currently used in clinical practice for treatment of active nAMD, demonstrating some beneficial effect in reducing related-subretinal fibrosis [103,104]. An open-label, safety phase II trial to study subretinal fibrosis in nAMD patients treated with intravitreal Fovista® (E10030), a platelet-derived growth factor (PDGF) antagonist, in combination with anti-VEGF therapy, has recently completed with published results [105].

Moreover, a multi-center, randomized, controlled phase II study assessing the change in subretinal fibrosis of intravitreal injections of RBM-007, a fibroblast growth factor 2 (FGF2) antagonist, as a monotherapy or in combination with intravitreal anti-VEGF therapy in nAMD, is currently active [106].

## 8. Biomechanical Factors

A crucial event during the wound healing process is the contraction of newly formed connective tissue by myofibroblasts. Myofibroblasts are highly contractile fibroblasts expressing α-smooth muscle actin (α-SMA), an actin isoform that contributes to cell-generation of mechanical tension. Myofibroblasts serve as a key role in tissue restoration as they provide both ECM components and mechanical strength to allow for wound contraction [107]. Wound contraction is necessary to reduce the size of the wound defect so that closure will occur. Myofibroblasts typically disband in apoptosis after successful repair. Persistent myofibroblast activation within the remodeling wound space can lead to stiff ECM collagen contractures, resulting in debilitating contractile scarring with dramatic consequences for organ function [107,108,109,110].

In an in vitro model of connective tissue remodeling, fibroblast-mediated contraction of collagen gels was reported in response to TGFβ [111,112]. Moreover, this process was observed to be strictly dependent on Smad3 [113,114]. Specifically, overexpression of Smad3 in the fibroblasts increased collagen gel contraction, while Smad7 overexpression reduced it [113]. Furthermore, TGFβ augmented contraction of Smad2 knockout (S2KO), Smad2 wildtype (S2WT), and Smad3 wildtype (S3WT), but not Smad3 knockout (S3KO) mouse fibroblasts [114]. Thus, the Smad3 gene may represent a target for blocking TGFβ-promoted fibrotic tissue contraction. 

In the cornea, stromal rigidity was identified as a critical factor in TGFβ-induced promotion of corneal epithelial wound healing in transwell and wound healing assay studies [24]. In vivo animal studies demonstrated elevation in the matrix stiffness of the anterior corneal stroma following wounding, and it correlated initially with the development of edema and inflammation, and later with stromal haze development and filling of the wound space with myofibroblasts [115]. Moreover, from the same study, stromal cells treated with TGFβ1 in vitro were stiffer than untreated cells, and their stiffness was significantly determined by the matrix stiffness of the corneal wound microenvironment [115]. Crosslinking (CXL) with riboflavin and hyaluronidase to stiffen or soften the corneal stroma, respectively, is an expanding strategy for modulating corneal biomechanics [115]. CXL is reported to promote anterior keratocyte apoptosis/necrosis and transformation of fibroblasts into myofibroblasts around the area of treatment [116]. CXL procedures are currently employed to treat degenerative corneal diseases.

Biomechanical properties also have relevance in glaucoma pathogenesis. In glaucoma, the LC, TM, and SC tissues demonstrated considerable ECM fibrotic changes and associated stiffness [117,118] upon stimulation with TGFβ [119] and cyclic stretch from in vivo and in vitro studies [120]. These remodeling events in the LC affected mechanical integrity, which in turn alters tissue response to chronic elevation of IOP. Stiffening in the LC was associated to progression of LC morphology from a normal state to that of a cupped, excavated glaucomatous state from computational and numerical modeling studies and mechanical testing of ocular tissues [121]. Increased stiffness of glaucomatous human TM and SC was correlated with an increased resistance to aqueous humor outflow and elevated IOP, thus influencing the onset and progression of glaucoma [121]. Moreover, the increased ocular rigidity in glaucoma was identified as a critical mechanical driver of the optic neuropathy as it influences outflow resistance and daily changes in IOP, from theoretical models and in vivo studies [122,123,124,125].

In addition, the filtering bleb after GFS is constantly subjected to mechanical stress induced by the draining of the aqueous humor. High levels of TGFβ2 were detected in the aqueous humor of patients with glaucoma [126], and TGFβ1 and β2 are also expressed in local cells (conjunctival epithelium and fibroblasts) in the filtering bleb tissue [35,127]. In a cellular model of conjunctival fibrosis, TGFβ2-Smad signaling was found to promote transcription of fibrotic genes in human primary conjunctival fibroblasts through interaction with the Yes-associated protein (YAP) and transcriptional coactivator with PDZ-binding motif (TAZ) in the Hippo pathway [128]. The Hippo pathway is the most representative mechanical stress-related signaling pathway. YAP and TAZ are key effectors of the Hippo pathway. In the presence of mechanical stress, YAP/TAZ are stabilized and translocate to the nucleus depending on TGFβ2, and subsequently interact with Smad2/3 transcription factors driving the transcription of fibrotic genes [129]. Verteporfin, a YAP/TAZ inhibitor, exerted potent antifibrosis effects by suppressing the TGFβ2-YAP/TAZ-Smad axis in human TM cells [128,130,131].

In various diseases, including PVR and nAMD, the retina is subjected to mechanical forces. Interestingly, substrates of varying elastic moduli were found to induce gene expression changes in rat Müller cells to promote expression of genes implicated in PVR [132]. In addition, a recent study demonstrated that matrix stiffness induced activation of ARPE-19 cells through the RhoA/YAP pathway and development of a retinal fibrogenesis in vivo PVR mouse model [133]. Interestingly, the upstream blockade of RhoA by C3 exoenzyme or downstream blockade of YAP by verteporfin effectively suppressed MMP expression in ARPE-19 cells and collagen gel contraction [133]. Furthermore, blockade of RhoA/YAP signaling reduced PVR-induced retinal fibrogenesis and inhibited the TGFβ/Smad pathway in vivo [133]. This suggests attractive novel therapeutic strategies targeting the RhoA/YAP pathway for treatment of PVR.

Biomechanical factors are also involved in the pathogenesis of nAMD. Specifically, changes in the biomechanical properties of the Bruch’s membrane were reported to affect diffusion of nutrients and/or waste between outer retina and choriocapillaris, possibly leading to AMD onset [134]. Moreover, a reduction of retinal elastin and, consequently, increased retinal stiffness was described in patients affected by moderate-to-severe AMD [135].

## 9. Cell-Based Therapies

The recent development of stem cell technologies holds promise for the treatment of diseases that are caused by cell degeneration or death, including fibrotic disorders to the eye. Cell-based therapies modulate the pathological responses in the eye by using rescue activities of the cells themselves or by delivering transgenes expressing molecules useful in the treatment of fibroproliferative disorders [136].

Autologous or allogeneic limbal epithelial stem cell transplantation is an accepted procedure for the treatment of limbal stem cell deficiency (LSCD) in ocular surface fibrotic disorders [137]. Limbal epithelial stem cells are transplanted into the eye of the diseased patient after collection using biopsy and expansion in culture [138].

However, the inability to obtain an autologous transplant in the case of bilateral disorders, as well as the need for immunosuppression after allogeneic grafting, forced investigations into an alternative cell source or therapy [139].

Transplantation of cultured oral mucosal epithelial cell sheets in patients with total LSCD resulted in complete stable epithelialization along with visual improvement [140].

Moreover, other than cornea, additional sources for stem cells in eye therapies include the bone marrow and umbilical cord [141]. Transplantation of bone marrow-derived stem cells resulted in being as equally efficient as limbal epithelial stem cell transplantation to improve corneal epithelial damage in LSCD [142]. Moreover, a recent study reported that subconjunctival injection of TNFα pre-stimulated bone marrow-derived stem cells enhanced anti-inflammatory and anti-fibrotic effect in ocular alkali burn in a rat model [143]. Moreover, bone marrow-derived stem cells, when injected directly in the eye, were demonstrated to selectively localize to activated glial cells, which are involved in many ocular retinal vascular and degenerative diseases [136]. The intravitreal administration of autologous bone marrow-derived CD34+ cells in patients with degenerative retinal conditions showed the incorporation of new cells into the macula of the study eye [144]. A number of clinical trials are currently investigating the efficacy and safety of bone marrow-derived stem cell transplantation in patients with ocular fibrotic degenerative diseases, including glaucoma, optic neuropathy, AMD, retinal diseases, and diabetic retinopathy [145,146,147,148,149]. However, results from a recent trial evaluating intravitreal injection of autologous bone marrow-derived stem cells in patients with advanced glaucoma reported no ERG response changes, no improvement in visual function, and significative side effects, suggesting further investigation [150].

Human umbilical cord stem cells also show potential in regeneration of eye tissues [151]. The effects of human umbilical cord stem cell-derived exosomes were investigated on subretinal fibrosis in vivo and in vitro [152]. Exosomes are nano-sized vesicles released from cells that deliver proteins, lipids, mRNA, and miRNA to neighboring or distant cells. Stem cell-derived exosomes showed potential in treatment ocular tissue fibrosis by transferring their molecular contents [153]. Intravitreal injection of human umbilical cord-derived exosomes effectively reduced subretinal fibrosis in vivo [152]. Moreover, exosomes can be loaded with therapeutic miRNA. Human umbilical cord stem cell-derived exosomal miR-27b repressed the EMT process in vitro RPE cells induced by TGFβ2 [154]. Moreover, a placebo-controlled, randomized, double-blind phase I/II trial is currently evaluating the efficacy and safety of umbilical cord stem cells injection in the treatment of corneal burn in human [155].

## 10. Conclusions

Ocular fibrosis is an irreversible process inevitably leading to blindness. Currently, there are no available therapies targeting the fibrotic process in the eye. The only available treatments include antimetabolite drugs that show significant potentially blinding side effects. Thus, there is an urgent need to identify novel therapeutic targets in ocular fibrosis. This review provides current and futures perspectives on different strategies for the treatment of fibrotic eye disorders. As is evident from the numerous studies discussed in this review, the increasing knowledge of TGFβ-regulated pathways, angiogenesis, and biomechanics in ocular fibrosis and the promising results obtained from novel therapies, especially in the area of gene therapy and cell-based therapy, could offer new perspectives in clinical practice in order to prevent/treat ocular fibrosis.

## Figures and Tables

**Table 1:** Preclinical studies with Gene transfer and non-gene transfer techniques targeting TGFβ/Smad in ocular fibrosis.

Target	Treatment Strategy	In Vitro and In Vivo Models	References
Gene transfer techniques			
TGFβ	Adenoviral gene transfer of soluble human type II TGFβ receptor	*In vitro in COS cells, in vivo in mouse alkali-burned cornea.*	*Sakamoto et al., Gene Ther. 2000*
TGFβ	siRNAs transfection of type II TGFβ receptor	*In vitro in cultured human corneal fibroblasts. In vivo in mouse model of ocular inflammation and fibrosis.*	*Nakamura et al., Mol Vis. 2004*
Smad3	Knocking out of Smad3 by gene targeting	*In vivo in mouse alkali-burned cornea.*	*Saika et al., Am. J. Pathol. 2005b*
Smad7	Adenoviral gene transfer of Smad7	*In vivo in mouse alkali-burned cornea.*	*Saika et al., Am. J. Pathol. 2005b*
		*In vitro in cultured human subconjunctival fibroblasts, in vivo in mouse injury-induced wound healing of conjunctiva.*	*Yamanaka et al., Mol Vis. 2006*
		*In vivo in mouse injured lens epithelium.*	*Saika et al., Lab Invest. 2004*
		*In vitro in ARPE-19 human RPE cells, in vivo PVR mouse model.*	*Saika et al., Arch Ophthalmol. 2007*
BMP-7	Adenoviral gene transfer of BMP-7	*In vivo in mouse alkali-burned cornea.* *In vitro in human corneal fibroblasts and myofibroblasts, in vivo in rabbit model of corneal fibrosis.*	*Saika et al., Lab Invest. 2005* *Gupta et al., Invest Ophthalmol Vis Sci. 2018*
BMP-7 Id2 Id3	Adenoviral gene transfer of BMP-7, Id2, or Id3	*In vivo in mouse injured lens epithelium.*	*Saika et al., Am J Physiol Cell Physiol. 2006*
PPARγ	Adenoviral gene transfer of PPARγ	*In vitro in mouse ocular fibroblasts and macrophages, and in human corneal epithelial cell line, in vivo in mouse alkali-burned cornea.*	*Saika et al., Am J Physiol Cell Physiol. 2007*
Decorin	Mammalian vector gene transfer of Decorin Adenoviral gene transfer of Decorin Adenoviral gene transfer of Decorin	*In vitro in human corneal fibroblasts.* *In vivo rabbit model of corneal fibrosis.* *In vivo rabbit model of corneal fibrosis.*	*Mohan et al., Exp Eye Res. 2010* *Mohan et al., Invest Ophthalmol Vis Sci. 2011* *Mohan et al., PLoS One. 2011*
Nox4	Adenoviral gene transfer of Ad-Nox4i	*In vitro in HTFs, in vivo in mouse model of GFS.*	*Shah et al., Antioxidants (Basel). 2020*
p38MAPK	Adenoviral gene transfer of DN p38MAPK	*In vivo in a mouse model of PVR.*	*Saika et al., Lab Invest. 2005*
Non-gene transfer techniques			
SN50	Inhibition of NF-κB	*In vivo in mouse alkali-burned cornea.*	*Saika et al., Am. J. Pathol. 2005c*
Disulfiram	Inhibition of ALDH	*In vitro in human OMMP fibroblasts, in vivo in mouse model of scarring AED.*	*Ahadome et al., JCI Insight 2016*
Halofuginone	Up-regulation of Smad7 and down-regulation of TβR-II	*In vitro in human corneal fibroblasts.*	*Nelson et al., Mol Vis. 2012*
Genipin	Inhibition of Smad2, p38 MAPK and CTGF	*In vitro in lens epithelial cell line alpha-TN4.*	*Kitano et al., J Cataract Refract Surg. 2006*
Emodin	Inhibition of TNFα	*In vitro in human subconjunctival fibroblasts, in vivo in mouse alkali-burned cornea.*	*Kitano et al., Invest Ophthalmol Vis Sci. 2007*
Tetrandrine	Upregulation of Smad7 and downregulation of Smad2	*In vitro in human subconjunctival fibroblasts.*	*Kitano et al., Curr Eye Res. 2008*
Pirfenidone	Inhibition of TGF-β1, β2, and β3	*In vitro in human ocular fibroblasts.*	*Stahnke et al., PLoS One. 2017*
CTGF	Inhibition of CTGF	*In vivo in a rabbit model of GFS.*	*Wang et al., Int. J. Ophthalmol. 2011*
AMA0526 Y-27632 NSC23766	Inhibition of Rho-kinase	*In vivo in a rabbit model of GFS.* *In vitro in HTFs, in vivo in a rabbit model of GFS.* *In vitro in ARPE-19 human RPE cells.* *In vitro in HTFs.*	*Van de Velde et al., Prog Brain Res. 2015* *Honjo et al., Invest Ophthalmol Vis Sci. 2007* *Zhu et al., Int J Ophthalmol. 2013* *Tovell et al., Invest Ophthalmol Vis Sci. 2012*
Sulforaphane LiCl 3-MA	Inhibition of PI3K/Akt	*In vitro in human conjunctival fibroblasts.* *In vitro in HTFs.* *In vivo in mouse model of subretinal fibrosis.*	*Liu et al., Int J Ophthalmol. 2020* *Chung et al., Biotechnol Lett. 2014* *Bo et al., J Ocul Pharmacol Ther. 2020*
SB203580 SB202190	Inhibition of p38MAPK	*In vitro in HTFs.* *In vitro in ARPE-19 human RPE cells.*	*Meyer-Ter-Vehn et al., Invest Ophthalmol Vis Sci. 2006* *Saika et al., Lab Invest. 2005*
RO4929097 LY411575 DAPT	Inhibition of Notch	*In vitro in human MIO-M1 Müller cells, in vivo in a murine model of retinal fibrosis.* *In vitro in RPE cells, in vivo in mouse model of PVR.* *In vitro in ARPE-19 human RPE cells.*	*Fan et al., Theranostics. 2020* *Zhang et al., Histochem Cell Biol. 2017* *Chen et al. Curr Mol Med. 2014*

TGFβ, transforming growth factor β; siRNAs, small interference RNAs; Smad, small mothers against decapentaplegic; RPE, retinal pigment epithelium; PVR, proliferative vitreoretinopathy; BMP-7, bone morphogenic protein-7; Id2 and Id3, inhibitors of differentiation 2 and 3; PPARγ, peroxisome proliferator-activated receptor γ; Nox4, NADPH oxidase 4; Ad-Nox4i, Nox4 small interfering RNA (siRNA); p38 MAPK, p38 mitogen-activated protein kinase; DN, dominant-negative; NF-κB, nuclear factor-κB; ALDH, aldehyde dehydrogenase; OMMP, ocular mucous membrane pemphigoid; AED, allergic eye disease; TβR-II, TGF-β receptor type II; TNFα, tumor necrosis factor α; CTGF, connective tissue growth factor; HTFs, human Tenon’s fibroblasts; GFS, glaucoma filtration surgery; PI3K/Akt, phosphatidylinositol-3-kinase/Akt; LiCl, lithium chloride; 3-MA, 3-methyladenine.

## References

[1] Friedlander M. (2007). Fibrosis and diseases of the eye. J. Clin. Investig..

[2] Saika S., Yamanaka O., Sumioka T., Miyamoto T., Miyazaki K.-I., Okada Y., Kitano A., Shirai K., Tanaka S.-I., Ikeda K. (2008). Fibrotic disorders in the eye: Targets of gene therapy. Prog. Retin. Eye Res..

[3] Chen M., Luo C., Zhao J., Devarajan G., Xu H. (2018). Immune regulation in the aging retina. Prog. Retin. Eye Res..

[4] Mochizuki M., Sugita S., Kamoi K. (2013). Immunological homeostasis of the eye. Prog. Retin. Eye Res..

[5] Pancholi S., Tullo A., Khaliq A., Foreman D., Boulton M. (1998). The effects of growth factors and conditioned media on the proliferation of human corneal epithelial cells and keratocytes. Graefe’s Arch. Clin. Exp. Ophthalmol..

[6] Barsam C.A., Brick D.J., Jones C., Wechsler S.L., Perng O. (2005). A Viral Model for Corneal Scarring and Neovascularization Following Ocular Infection of Rabbits with a Herpes Simplex Virus Type 1 (HSV-1) Mutant. Cornea.

[7] Menko A.S., Walker J.L., Stepp M.A. (2019). Fibrosis: Shared Lessons From the Lens and Cornea. Anat. Rec. Adv. Integr. Anat. Evol. Biol..

[8] Faraj H.G., Hoang-Xuan T. (2001). Chronic cicatrizing conjunctivitis. Curr. Opin. Ophthalmol..

[9] Wallace D., Pokrovskaya O., O’Brien C.J. (2015). The Function of Matricellular Proteins in the Lamina Cribrosa and Trabecular Meshwork in Glaucoma. J. Ocul. Pharmacol. Ther..

[10] Khaw P.T., Chiang M., Shah P., Sii F., Lockwood A., Khalili A. (2017). Enhanced Trabeculectomy: The Moorfields Safer Surgery System. Dev. Ophthalmol..

[11] Lovicu F., Shin E., McAvoy J. (2015). Fibrosis in the lens. Sprouty regulation of TGFβ-signaling prevents lens EMT leading to cataract. Exp. Eye Res..

[12] Abu El-Asrar A.M., Nawaz M.I., Ahmad A., Siddiquei M.M., Allegaert E., Gikandi P.W., De Hertogh G., Opdenakker G. (2021). CD146/Soluble CD146 Pathway Is a Novel Biomarker of Angiogenesis and Inflammation in Proliferative Diabetic Retinopathy. Investig. Opthalmology Vis. Sci..

[13] Singh A., Faber C., Falk M., Nissen M.H., Hviid T.V., Sørensen T.L. (2012). Altered Expression of CD46 and CD59 on Leukocytes in Neovascular Age-Related Macular Degeneration. Am. J. Ophthalmol..

[14] Chaudhary R., Scott R.A.H., Wallace G., Berry M., Logan A., Blanch R.J. (2020). Inflammatory and Fibrogenic Factors in Proliferative Vitreoretinopathy Development. Transl. Vis. Sci. Technol..

[15] Saika S., Yamanaka O., Flanders K.C., Okada Y., Miyamoto T., Sumioka T., Shirai K., Kitano A., Miyazaki K.-I., Tanaka S.-I. (2008). Epithelial-Mesenchymal Transition as a Therapeutic Target for Prevention of Ocular Tissue Fibrosis. Endocr. Metab. Immune Disord.-Drug Targets.

[16] Shu D.Y., Lovicu F.J. (2017). Myofibroblast transdifferentiation: The dark force in ocular wound healing and fibrosis. Prog. Retin. Eye Res..

[17] Saika S. (2005). TGFβ pathobiology in the eye. Lab. Investig..

[18] Saika S. (2009). TGFb in fibroproliferative diseases in the eye. Front. Biosci..

[19] Finnson K.W., McLean S., Di Guglielmo G.M., Philip A. (2013). Dynamics of Transforming Growth Factor Beta Signaling in Wound Healing and Scarring. Adv. Wound Care.

[20] Karamichos D., Hjortdal J. (2014). Keratoconus: Tissue Engineering and Biomaterials. J. Funct. Biomater..

[21] Matsuba M., Hutcheon A.E., Zieske J.D. (2011). Localization of thrombospondin-1 and myofibroblasts during corneal wound repair. Exp. Eye Res..

[22] Tzavlaki K., Moustakas A. (2020). TGF-β Signaling. Biomolecules.

[23] Miyazawa K., Miyazono K. (2016). Regulation of TGF-β Family Signaling by Inhibitory Smads. Cold Spring Harb. Perspect. Biol..

[24] Yang Y.-H., Hsieh T.-L., Ji A.T.-Q., Hsu W.-T., Liu C.-Y., Lee O.K.-S., Ho J.H.-C. (2016). Stromal Tissue Rigidity Promotes Mesenchymal Stem Cell-Mediated Corneal Wound Healing Through the Transforming Growth Factor β Signaling Pathway. Stem Cells.

[25] Piek E., Ju W.J., Heyer J., Escalante-Alcalde D., Stewart C.L., Weinstein M., Deng C., Kucherlapati R., Böttinger E.P., Roberts A.B. (2001). Functional Characterization of Transforming Growth Factor β Signaling in Smad2- and Smad3-deficient Fibroblasts. J. Biol. Chem..

[26] Roberts A., Tian F., Byfield S., Stuelten C., Ooshima A., Saika S., Flanders K. (2006). Smad3 is key to TGF-β-mediated epithelial-to-mesenchymal transition, fibrosis, tumor suppression and metastasis. Cytokine Growth Factor Rev..

[27] Li J., Tang X., Chen X. (2011). Comparative effects of TGF-β2/Smad2 and TGF-β2/Smad3 signaling pathways on proliferation, migration, and extracellular matrix production in a human lens cell line. Exp. Eye Res..

[28] Meng F., Li J., Yang X., Yuan X., Tang X. (2018). Role of Smad3 signaling in the epithelial-mesenchymal transition of the lens epithelium following injury. Int. J. Mol. Med..

[29] Saika S., Kono-Saika S., Tanaka T., Yamanaka O., Ohnishi Y., Sato M., Muragaki Y., Ooshima A., Yoo J., Flanders K.C. (2004). Smad3 is required for dedifferentiation of retinal pigment epithelium following retinal detachment in mice. Lab. Investig..

[30] Saika S., Kono-Saika S., Ohnishi Y., Sato M., Muragaki Y., Ooshima A., Flanders K.C., Yoo J., Anzano M., Liu C.-Y. (2004). Smad3 Signaling Is Required for Epithelial-Mesenchymal Transition of Lens Epithelium after Injury. Am. J. Pathol..

[31] Stramer B.M., Austin J.S., Roberts A.B., Fini M.E. (2004). Selective reduction of fibrotic markers in repairing corneas of mice deficient in Smad3. J. Cell. Physiol..

[32] Saika S., Ikeda K., Yamanaka O., Flanders K.C., Okada Y., Miyamoto T., Kitano A., Ooshima A., Nakajima Y., Ohnishi Y. (2006). Loss of Tumor Necrosis Factor α Potentiates Transforming Growth Factor β-mediated Pathogenic Tissue Response during Wound Healing. Am. J. Pathol..

[33] Fujita S., Saika S., Kao W.W.-Y., Fujita K., Miyamoto T., Ikeda K., Nakajima Y., Ohnishi Y. (2007). Endogenous TNFα Suppression of Neovascularization in Corneal Stroma in Mice. Investig. Opthalmology Vis. Sci..

[34] Ulloa L., Doody J.F., Massague J. (1999). Inhibition of transforming growth factor-β/SMAD signalling by the interferon-γ/STAT pathway. Nature.

[35] Yamanaka O., Saika S., Okada Y., Ooshima A., Ohnishi Y. (2003). Effects of interferon-γ on human subconjunctival fibroblasts in the presence of TGFβ1: Reversal of TGFβ-stimulated collagen production. Graefe’s Arch. Clin. Exp. Ophthalmol..

[36] Yuan H.-P., Li X.-H., Yang B.-B., Shao Z.-B., Yan L.-P. (2009). Expression of connective tissue growth factor after trabeculectomy in rabbits. Chin. J. Ophthalmol..

[37] Wang Y., Chang T., Wu T., Ye W., Wang Y., Dou G., Du H., Hui Y., Guo C. (2021). Connective tissue growth factor promotes retinal pigment epithelium mesenchymal transition via the PI3K/AKT signaling pathway. Mol. Med. Rep..

[38] Micera A., Lambiase A., Stampachiacchiere B., Bonini S., Levi-Schaffer F. (2007). Nerve growth factor and tissue repair remodeling: trkANGFR and p75NTR, two receptors one fate. Cytokine Growth Factor Rev..

[39] Micera A., Stampachiacchiere B., Di Zazzo A., Sgrulletta R., Cortés M., Normando E.M., Lambiase A., Bonini S. (2015). NGF Modulates trkANGFR/p75NTR in αSMA-Expressing Conjunctival Fibroblasts from Human Ocular Cicatricial Pemphigoid (OCP). PLoS ONE.

[40] Biernacka A., Dobaczewski M., Frangogiannis N.G. (2011). TGF-β signaling in fibrosis. Growth Factors.

[41] Cargnello M., Roux P.P. (2011). Activation and Function of the MAPKs and Their Substrates, the MAPK-Activated Protein Kinases. Microbiol. Mol. Biol. Rev..

[42] Kim S.-J., Kim Y.-S., Kim J.H., Jang H.Y., Da Ly D., Das R., Park K.-S. (2020). Activation of ERK1/2-mTORC1-NOX4 mediates TGF-β1-induced epithelial-mesenchymal transition and fibrosis in retinal pigment epithelial cells. Biochem. Biophys. Res. Commun..

[43] Okada Y., Saika S., Shirai K., Yamanaka O., Kitano A., Wang Z., Yang H., Reinach P. (2009). JNK MAPK Signaling Contributes in vivo to Injury-Induced Corneal Epithelial Migration. Ophthalmic Res..

[44] Joko T., Shiraishi A., Akune Y., Tokumaru S., Kobayashi T., Miyata K., Ohashi Y. (2012). Involvement of P38MAPK in human corneal endothelial cell migration induced by TGF-β(2). Exp. Eye Res..

[45] Kimoto K., Nakatsuka K., Matsuo N., Yoshioka H. (2004). p38 MAPK Mediates the Expression of Type I Collagen Induced by TGF-β2 in Human Retinal Pigment Epithelial Cells ARPE-19. Investig. Opthalmology Vis. Sci..

[46] Itoh Y., Kimoto K., Imaizumi M., Nakatsuka K. (2007). Inhibition of RhoA/Rho-kinase pathway suppresses the expression of type I collagen induced by TGF-β2 in human retinal pigment epithelial cells. Exp. Eye Res..

[47] Yu-Wai-Man C., Treisman R., Bailly M., Khaw P.T. (2014). The Role of the MRTF-A/SRF Pathway in Ocular Fibrosis. Investig. Opthalmology Vis. Sci..

[48] Gupta M., Korol A., West-Mays J.A. (2013). Nuclear translocation of myocardin-related transcription factor—A during transforming growth factor beta-induced epithelial to mesenchymal transition of lens epithelial cells. Mol. Vis..

[49] Yang Y.-C., Piek E., Zavadil J., Liang D., Xie D., Heyer J., Pavlidis P., Kucherlapati R., Roberts A.B., Böttinger E.P. (2003). Hierarchical model of gene regulation by transforming growth factor. Proc. Natl. Acad. Sci. USA.

[50] Shi Y., Massagué J. (2003). Mechanisms of TGF-β Signaling from Cell Membrane to the Nucleus. Cell.

[51] Massagué J., Gomis R. (2006). The logic of TGFβ signaling. FEBS Lett..

[52] Yokoyama K., Kimoto K., Itoh Y., Nakatsuka K., Matsuo N., Yoshioka H., Kubota T. (2011). The PI3K/Akt pathway mediates the expression of type I collagen induced by TGF-β2 in human retinal pigment epithelial cells. Graefe’s Arch. Clin. Exp. Ophthalmol..

[53] Sakamoto T., Ueno H., Sonoda K., Hisatomi T., Shimizu K., Ohashi H., Inomata H. (2000). Blockade of TGF-β by in vivo gene transfer of a soluble TGF-β type II receptor in the muscle inhibits corneal opacification, edema and angiogenesis. Gene Ther..

[54] Nakamura H., Siddiqui S.S., Shen X., Malik A.B., Pulido J.S., Kumar N.M., Yue B.Y. (2004). RNA interference targeting transforming growth factor-beta type II receptor suppresses ocular inflammation and fibrosis. Mol. Vis..

[55] Saika S. (2004). TGF-β Signal Transduction in Corneal Wound Healing as a Therapeutic Target. Cornea.

[56] Saika S., Okada Y., Miyamoto T., Yamanaka O., Ohnishi Y., Ooshima A., Liu C.-Y., Weng D., Kao W.W.-Y. (2004). Role of p38 MAP Kinase in Regulation of Cell Migration and Proliferation in Healing Corneal Epithelium. Investig. Opthalmology Vis. Sci..

[57] Flanders K.C. (2004). Smad3 as a mediator of the fibrotic response. Int. J. Exp. Pathol..

[58] Saika S., Ikeda K., Yamanaka O., Miyamoto T., Ohnishi Y., Sato M., Muragaki Y., Ooshima A., Nakajima Y., Kao W.W.-Y. (2005). Expression of Smad7 in Mouse Eyes Accelerates Healing of Corneal Tissue after Exposure to Alkali. Am. J. Pathol..

[59] Yamanaka O., Ikeda K., Saika S., Miyazaki K., Akira Ooshima Y.O. (2006). Gene transfer of Smad7 modulates injury-induced conjunctival wound healing in mice. molvis.org.undefined. Gene transfer of Smad7 modulates injury-induced conjunctival wound healing in mice. Mol. Vis..

[60] Saika S., Ikeda K., Yamanaka O., Sato M., Muragaki Y., Ohnishi Y., Ooshima A., Nakajima Y., Namikawa K., Kiyama H. (2004). Transient adenoviral gene transfer of Smad7 prevents injury-induced epithelial–mesenchymal transition of lens epithelium in mice. Lab. Investig..

[61] Saika S., Yamanaka O., Nishikawa-Ishida I., Kitano A., Flanders K.C., Okada Y., Ohnishi Y., Nakajima Y., Ikeda K. (2007). Effect of Smad7 Gene Overexpression on Transforming Growth Factor β–Induced Retinal Pigment Fibrosis in a Proliferative Vitreoretinopathy Mouse Model. Arch. Ophthalmol..

[62] Saika S., Ikeda K., Yamanaka O., Flanders K.C., Nakajima Y., Miyamoto T., Ohnishi Y., Kao W.W.-Y., Muragaki Y., Ooshima A. (2005). Therapeutic effects of adenoviral gene transfer of bone morphogenic protein-7 on a corneal alkali injury model in mice. Lab. Investig..

[63] Saika S., Ikeda K., Yamanaka O., Flanders K.C., Ohnishi Y., Nakajima Y., Muragaki Y., Ooshima A. (2006). Adenoviral gene transfer of BMP-7, Id2, or Id3 suppresses injury-induced epithelial-to-mesenchymal transition of lens epithelium in mice. Am. J. Physiol. Physiol..

[64] Gupta S., Fink M.K., Ghosh A., Tripathi R., Sinha P.R., Sharma A., Hesemann N.P., Chaurasia S., Giuliano E.A., Mohan R.R. (2018). Novel Combination BMP7 and HGF Gene Therapy Instigates Selective Myofibroblast Apoptosis and Reduces Corneal Haze In Vivo. Investig. Opthalmology Vis. Sci..

[65] Chinetti G., Fruchart J.-C., Staels B. (2000). Peroxisome proliferator-activated receptors (PPARs): Nuclear receptors at the crossroads between lipid metabolism and inflammation. Inflamm. Res..

[66] Moraes L.A., Piqueras L., Bishop-Bailey D. (2006). Peroxisome proliferator-activated receptors and inflammation. Pharmacol. Ther..

[67] Saika S., Yamanaka O., Okada Y., Miyamoto T., Kitano A., Flanders K.C., Ohnishi Y., Nakajima Y., Kao W.W.-Y., Ikeda K. (2007). Effect of overexpression of pparγ on the healing process of corneal alkali burn in mice. Am. J. Physiol. Physiol..

[68] Mohan R.R., Gupta R., Mehan M.K., Cowden J.W., Sinha S. (2010). Decorin transfection suppresses profibrogenic genes and myofibroblast formation in human corneal fibroblasts. Exp. Eye Res..

[69] Mohan R.R., Tandon A., Sharma A., Cowden J.W., Tovey J.C.K. (2011). Significant Inhibition of Corneal Scarring In Vivo with Tissue-Selective, Targeted AAV5 Decorin Gene Therapy. Investig. Opthalmology Vis. Sci..

[70] Mohan R.R., Tovey J.C.K., Sharma A., Schultz G.S., Cowden J.W., Tandon A. (2011). Targeted Decorin Gene Therapy Delivered with Adeno-Associated Virus Effectively Retards Corneal Neovascularization In Vivo. PLoS ONE.

[71] Shah M.H., Chan E.C., Van Bergen N.J., Pandav S.S., Ng S., Crowston J.G., Peshavariya H.M. (2020). Nox4 Facilitates TGFβ1-Induced Fibrotic Response in Human Tenon’s Fibroblasts and Promotes Wound Collagen Accumulation in Murine Model of Glaucoma Filtration Surgery. Antioxidants.

[72] Saika S., Yamanaka O., Ikeda K., Kim-Mitsuyama S., Flanders K.C., Yoo J., Roberts A.B., Nishikawa-Ishida I., Ohnishi Y., Muragaki Y. (2005). Inhibition of p38MAP kinase suppresses fibrotic reaction of retinal pigment epithelial cells. Lab. Investig..

[73] Saika S., Miyamoto T., Yamanaka O., Kato T., Ohnishi Y., Flanders K.C., Ikeda K., Nakajima Y., Kao W.W.-Y., Sato M. (2005). Therapeutic Effect of Topical Administration of SN50, an Inhibitor of Nuclear Factor-κB, in Treatment of Corneal Alkali Burns in Mice. Am. J. Pathol..

[74] Xu X., Li Y.N., Chen C.W., Trinh-Minh T., Schett G., Distler J.H.W. (2021). POS0327 INACTIVATION OF ALDEHYDE DEHYDROGENASE 3A2 INHIBITS FIBROBLAST ACTIVATION AND TISSUE FIBROSIS. Ann. Rheum. Dis..

[75] Ahadome S.D., Abraham D.J., Rayapureddi S., Saw V.P., Saban D.R., Calder V.L., Norman J.T., Ponticos M., Daniels J.T., Dart J.K. (2016). Aldehyde dehydrogenase inhibition blocks mucosal fibrosis in human and mouse ocular scarring. JCI Insight.

[76] Xavier S., Piek E., Fujii M., Javelaud D., Mauviel A., Flanders K.C., Samuni A.M., Felici A., Reiss M., Yarkoni S. (2004). Amelioration of Radiation-induced Fibrosis. J. Biol. Chem..

[77] Nelson E.F., Huang C.W., Ewel J.M., Chang A.A., Yuan C. (2012). Halofuginone down-regulates Smad3 expression and inhibits the TGFbeta-induced expression of fibrotic markers in human corneal fibroblasts. Mol. Vis..

[78] Kitano A., Saika S., Yamanaka O., Reinach P.S., Ikeda K., Okada Y., Shirai K., Ohnishi Y. (2006). Genipin suppression of fibrogenic behaviors of the α-TN4 lens epithelial cell line. J. Cataract. Refract. Surg..

[79] Kitano A., Saika S., Yamanaka O., Ikeda K., Okada Y., Shirai K., Reinach P.S. (2007). Emodin Suppression of Ocular Surface Inflammatory Reaction. Investig. Opthalmology Vis. Sci..

[80] Kitano A., Yamanaka O., Ikeda K., Ishida-Nishikawa I., Okada Y., Shirai K., Saika S. (2008). Tetrandrine Suppresses Activation of Human Subconjunctival Fibroblasts In Vitro. Curr. Eye Res..

[81] Stahnke T., Kowtharapu B.S., Stachs O., Schmitz K.-P., Wurm J., Wree A., Guthoff R.F., Hovakimyan M. (2017). Suppression of TGF-β pathway by pirfenidone decreases extracellular matrix deposition in ocular fibroblasts in vitro. PLoS ONE.

[82] Wang J.-M., Hui N., Fan Y.-Z., Xiong L., Sun N.-X. (2011). Filtering bleb area and intraocular pressure following subconjunctival injection of CTGF antibody after glaucoma filtration surgery in rabbits. Int. J. Ophthalmol..

[83] Van de Velde S., Van Bergen T., Vandewalle E., Kindt N., Castermans K., Moons L., Stalmans I. (2015). Rho kinase inhibitor AMA0526 improves surgical outcome in a rabbit model of glaucoma filtration surgery. Prog. Brain Res..

[84] Honjo M., Tanihara H., Kameda T., Kawaji T., Yoshimura N., Araie M. (2007). Potential Role of Rho-Associated Protein Kinase Inhibitor Y-27632 in Glaucoma Filtration Surgery. Investig. Opthalmology Vis. Sci..

[85] Tovell V.E., Chau C.Y., Khaw P.T., Bailly M. (2012). Rac1 Inhibition Prevents Tissue Contraction and MMP Mediated Matrix Remodeling in the Conjunctiva. Investig. Opthalmology Vis. Sci..

[86] Zhu J., Nguyen D., Ouyang H., Zhang X.-H., Chen X.-M., Zhang K. (2013). Inhibition of RhoA/Rho-kinase pathway suppresses the expression of extracellular matrix induced by CTGF or TGF-β in ARPE-19. Int. J. Ophthalmol..

[87] Liu H.-R., Xia Z.-Y., Wang N.-L. (2020). Sulforaphane modulates TGFβ2-induced conjunctival fibroblasts activation and fibrosis by inhibiting PI3K/Akt signaling. Int. J. Ophthalmol..

[88] Chung E.J., Sohn Y.H., Kwon S.H., Jung S.-A., Lee J.H. (2014). Lithium chloride inhibits TGF-β1-induced myofibroblast transdifferentiation via PI3K/Akt pathway in cultured fibroblasts from Tenon’s capsule of the human eye. Biotechnol. Lett..

[89] Bo Q., Shen M., Xiao M., Liang J., Zhai Y., Zhu H., Jiang M., Wang F., Luo X., Sun X. (2020). 3-Methyladenine Alleviates Experimental Subretinal Fibrosis by Inhibiting Macrophages and M2 Polarization Through the PI3K/Akt Pathway. J. Ocul. Pharmacol. Ther..

[90] Meyer-Ter-Vehn T., Gebhardt S., Sebald W., Buttmann M., Grehn F., Schlunck G., Knaus P. (2006). p38 Inhibitors Prevent TGF-β–Induced Myofibroblast Transdifferentiation in Human Tenon Fibroblasts. Investig. Opthalmology Vis. Sci..

[91] Zhang J., Yuan G., Dong M., Zhang T., Hua G., Zhou Q., Shi W. (2016). Notch signaling modulates proliferative vitreoretinopathy via regulating retinal pigment epithelial-to-mesenchymal transition. Histochem. Cell Biol..

[92] Fan J., Shen W., Lee S.-R., Mathai A.E., Zhang R., Xu G., Gillies M.C. (2020). Targeting the Notch and TGF-β signaling pathways to prevent retinal fibrosis in vitro and in vivo. Theranostics.

[93] Chen X., Xiao W., Liu X., Zeng M., Luo L., Wu M., Ye S., Liu Y. (2014). Blockade of Jagged/Notch Pathway Abrogates Transforming Growth Factor β2-Induced Epithelial-Mesenchymal Transition in Human Retinal Pigment Epithelium Cells. Curr. Mol. Med..

[94] Khaw P., Grehn F., Holló G., Overton B., Wilson R., Vogel R.A., Smith Z.D.J. (2007). A Phase III Study of Subconjunctival Human Anti–Transforming Growth Factor β2 Monoclonal Antibody (CAT-152) to Prevent Scarring after First-Time Trabeculectomy. Ophthalmology.

[95] Search of: NCT02599064-List Results-ClinicalTrials.gov. NCT02599064.

[96] Bharadwaj A.S., Appukuttan B., Wilmarth P.A., Pan Y., Stempel A.J., Chipps T.J., Benedetti E.E., Zamora D.O., Choi D., David L.L. (2013). Role of the retinal vascular endothelial cell in ocular disease. Prog. Retin. Eye Res..

[97] Cho Y.K., Zhang X., Uehara H., Young J.R., Archer B., Ambati B. (2012). Vascular Endothelial Growth Factor Receptor 1 Morpholino Increases Graft Survival in a Murine Penetrating Keratoplasty Model. Investig. Opthalmology Vis. Sci..

[98] Vahedian Z., Mafi M., Fakhraie G., Zarei R., Eslami Y., Ghadimi H., Mohebbi M. (2017). Short-term Results of Trabeculectomy Using Adjunctive Intracameral Bevacizumab Versus Mitomycin C: A Randomized Controlled Trial. J. Glaucoma.

[99] Vandewalle E., Pinto L.A., Van Bergen T., Spielberg L., Fieuws S., Moons L., Spileers W., Zeyen T., Stalmans I. (2013). Intracameral bevacizumab as an adjunct to trabeculectomy: A 1-year prospective, randomised study. Br. J. Ophthalmol..

[100] Rabina G., Barequet D., Mimouni M., Kurtz S., Shemesh G., Rosenblatt A., Rosenfeld E. (2019). Intracameral bevacizumab role in trabeculectomy: A 1-year prospective randomized controlled study. Eur. J. Ophthalmol..

[101] Van Bergen T., Jonckx B., Hollanders K., Sijnave D., Van de Velde S., Vandewalle E., Moons L., Stassen J., Stalmans I. (2013). Inhibition of placental growth factor improves surgical outcome of glaucoma surgery. J. Cell. Mol. Med..

[102] Van de Veire S., Stalmans I., Heindryckx F., Oura H., Tijeras-Raballand A., Schmidt T., Loges S., Albrecht I., Jonckx B., Vinckier S. (2010). Further Pharmacological and Genetic Evidence for the Efficacy of PlGF Inhibition in Cancer and Eye Disease. Cell.

[103] Daniel E., Ying G.-S., Kim B.J., Toth C.A., Ferris F., Martin D.F., Grunwald J.E., Jaffe G.J., Dunaief J.L., Pan W. (2019). Five-Year Follow-up of Nonfibrotic Scars in the Comparison of Age-Related Macular Degeneration Treatments Trials. Ophthalmology.

[104] Kaiser P.K., Blodi B.A., Shapiro H., Acharya N.R. (2007). Angiographic and Optical Coherence Tomographic Results of the MARINA Study of Ranibizumab in Neovascular Age-Related Macular Degeneration. Ophthalmology.

[105] Search of: NCT02214628—List Results—ClinicalTrials.gov [Internet]. NCT02214628.

[106] Search of: NCT04200248—List Results—ClinicalTrials.gov [Internet]. NCT04200248.

[107] Shinde A.V., Humeres C., Frangogiannis N.G. (2016). The role of α-smooth muscle actin in fibroblast-mediated matrix contraction and remodeling. Biochim. Biophys. Acta (BBA)-Mol. Basis Dis..

[108] Rocher M., Robert P.-Y., Desmoulière A. (2019). The myofibroblast, biological activities and roles in eye repair and fibrosis. A focus on healing mechanisms in avascular cornea. Eye.

[109] Hinz B. (2016). The role of myofibroblasts in wound healing. Curr. Res. Transl. Med..

[110] Hinz B. (2016). Myofibroblasts. Exp. Eye Res..

[111] Montesano R., Orci L. (1988). Transforming growth factor beta stimulates collagen-matrix contraction by fibroblasts: Implications for wound healing. Proc. Natl. Acad. Sci. USA.

[112] Grinnell F. (1994). Fibroblasts, myofibroblasts, and wound contraction. J. Cell Biol..

[113] Sumiyoshi K., Nakao A., Setoguchi Y., Okumura K., Tsuboi R., Ogawa H. (2003). Smads regulate collagen gel contraction by human dermal fibroblasts. Br. J. Dermatol..

[114] Liu X., Wen F.-Q., Kobayashi T., Abe S., Fang Q., Piek E., Bottinger E.P., Roberts A.B., Rennard S.I. (2003). Smad3 mediates the TGF-?-induced contraction of type I collagen gels by mouse embryo fibroblasts. Cell Motil. Cytoskelet..

[115] Raghunathan V.K., Thomasy S.M., Strøm P., Yañez-Soto B., Garland S.P., Sermeno J., Reilly C.M., Murphy C.J. (2017). Tissue and cellular biomechanics during corneal wound injury and repair. Acta Biomater..

[116] Esquenazi S., He J., Li N., Bazan H.E.P. (2010). Immunofluorescence of Rabbit Corneas After Collagen Cross-Linking Treatment with Riboflavin and Ultraviolet A. Cornea.

[117] Tamm E.R., Braunger B.M., Fuchshofer R. (2015). Intraocular Pressure and the Mechanisms Involved in Resistance of the Aqueous Humor Flow in the Trabecular Meshwork Outflow Pathways. Prog. Mol. Biol. Transl. Sci..

[118] Hopkins A., Murphy R., Irnaten M., Wallace D.M., Quill B., O’Brien C. (2020). The role of lamina cribrosa tissue stiffness and fibrosis as fundamental biomechanical drivers of pathological glaucoma cupping. Am. J. Physiol. Physiol..

[119] Kirwan R.P., Leonard M.O., Murphy M., Clark A.F., O’Brien C.J. (2005). Transforming growth factor-β-regulated gene transcription and protein expression in human GFAP-negative lamina cribrosa cells. Glia.

[120] Kirwan R.P., Crean J.K., Fenerty C.H., Clark A.F., O’Brien C.J. (2004). Effect of Cyclical Mechanical Stretch and Exogenous Transforming Growth Factor-??1 on Matrix Metalloproteinase-2 Activity in Lamina Cribrosa Cells from the Human Optic Nerve Head. J. Glaucoma.

[121] Liu B., McNally S., Kilpatrick J., Jarvis S.P., O’Brien C.J. (2018). Aging and ocular tissue stiffness in glaucoma. Surv. Ophthalmol..

[122] Ebneter A., Wagels B., Zinkernagel M.S. (2008). Non-invasive biometric assessment of ocular rigidity in glaucoma patients and controls. Eye.

[123] Hommer A., Fuchsja¨ger-Mayrl G., Resch H., Vass C., Garhofer G., Schmetterer L. (2008). Estimation of Ocular Rigidity Based on Measurement of Pulse Amplitude Using Pneumotonometry and Fundus Pulse Using Laser Interferometry in Glaucoma. Investig. Opthalmology Vis. Sci..

[124] Campbell I., Coudrillier B., Ethier C.R. (2014). Biomechanics of the Posterior Eye: A Critical Role in Health and Disease. J. Biomech. Eng..

[125] Burgoyne C., Downs J.C., Bellezza A.J., Suh J.-K.F., Hart R.T. (2005). The optic nerve head as a biomechanical structure: A new paradigm for understanding the role of IOP-related stress and strain in the pathophysiology of glaucomatous optic nerve head damage. Prog. Retin. Eye Res..

[126] Tripathi R.C., Li J., Chan W.A., Tripathi B.J. (1994). Aqueous Humor in Glaucomatous Eyes Contains an Increased Level of TGF-β 2. Exp. Eye Res..

[127] Saika S., Yamanaka O., Baba Y., Kawashima Y., Shirai K., Miyamoto T., Okada Y., Ohnishi Y., Ooshima A. (2001). Accumulation of latent transforming growth factor-β binding protein-1 and TGFβ1 in extracellular matrix of filtering bleb and of cultured human subconjunctival fibroblasts. Graefe’s Arch. Clin. Exp. Ophthalmol..

[128] Futakuchi A., Inoue T., Wei F.-Y., Inoue-Mochita M., Fujimoto T., Tomizawa K., Tanihara H. (2018). YAP/TAZ Are Essential for TGF-β2–Mediated Conjunctival Fibrosis. Investig. Opthalmology Vis. Sci..

[129] Szeto S.G., Narimatsu M., Lu M., He X., Sidiqi A.M., Tolosa M.F., Chan L., De Freitas K., Bialik J.F., Majumder S. (2016). YAP/TAZ Are Mechanoregulators of TGF-β-Smad Signaling and Renal Fibrogenesis. J. Am. Soc. Nephrol..

[130] Yemanyi F., Raghunathan V. (2020). Lysophosphatidic Acid and IL-6 Trans-signaling Interact via YAP/TAZ and STAT3 Signaling Pathways in Human Trabecular Meshwork Cells. Investig. Opthalmology Vis. Sci..

[131] Chen W.-S., Cao Z., Krishnan C., Panjwani N. (2015). Verteporfin without light stimulation inhibits YAP activation in trabecular meshwork cells: Implications for glaucoma treatment. Biochem. Biophys. Res. Commun..

[132] Davis J.T., Wen Q., Janmey P.A., Otteson D.C., Foster W.J. (2012). Müller Cell Expression of Genes Implicated in Proliferative Vitreoretinopathy Is Influenced by Substrate Elastic Modulus. Investig. Opthalmology Vis. Sci..

[133] Zhang W., Han H. (2021). Targeting matrix stiffness-induced activation of retinal pigment epithelial cells through the RhoA/YAP pathway ameliorates proliferative vitreoretinopathy. Exp. Eye Res..

[134] Chirco K.R., Sohn E., Stone E.M., Tucker B., Mullins R.F. (2016). Structural and molecular changes in the aging choroid: Implications for age-related macular degeneration. Eye.

[135] Chen K., Weiland J.D. (2013). Discovery of Retinal Elastin and Its Possible Role in Age-Related Macular Degeneration. Ann. Biomed. Eng..

[136] Otani A., Kinder K., Ewalt K.L., Otero F.J., Schimmel P., Friedlander M. (2002). Bone marrow–derived stem cells target retinal astrocytes and can promote or inhibit retinal angiogenesis. Nat. Med..

[137] Sacchetti M., Rama P., Bruscolini A., Lambiase A. (2018). Limbal Stem Cell Transplantation: Clinical Results, Limits, and Perspectives. Stem Cells Int..

[138] Sacchetti M., Lambiase A., Cortes M., Sgrulletta R., Bonini S., Merlo D., Bonini S. (2005). Clinical and cytological findings in limbal stem cell deficiency. Graefe’s Arch. Clin. Exp. Ophthalmol..

[139] Gurusamy N., Alsayari A., Rajasingh S., Rajasingh J. (2018). Adult Stem Cells for Regenerative Therapy. Prog. Mol. Biol. Transl. Sci..

[140] Kim Y.J., Lee H.J., Ryu J.S., Kim Y.H., Jeon S., Oh J.Y., Choung H.K., Khwarg S.I., Wee W.R., Kim M.K. (2017). Prospective Clinical Trial of Corneal Reconstruction with Biomaterial-Free Cultured Oral Mucosal Epithelial Cell Sheets. Cornea.

[141] Samoila O., Samoila L. (2021). Stem Cells in the Path of Light, from Corneal to Retinal Reconstruction. Biomedicines.

[142] Calonge M., Pérez I., Galindo S., Nieto-Miguel T., López-Paniagua M., Fernández I., Alberca M., García-Sancho J., Sánchez A., Herreras J.M. (2018). A proof-of-concept clinical trial using mesenchymal stem cells for the treatment of corneal epithelial stem cell deficiency. Transl. Res..

[143] Zhang N., Luo X., Zhang S., Liu R., Liang L., Su W., Liang D. (2020). Subconjunctival injection of tumor necrosis factor-α pre-stimulated bone marrow-derived mesenchymal stem cells enhances anti-inflammation and anti-fibrosis in ocular alkali burns. Graefe’s Arch. Clin. Exp. Ophthalmol..

[144] Park S.S., Bauer G., Abedi M., Pontow S., Panorgias A., Jonnal R.S., Zawadzki R., Werner J.S., Nolta J. (2014). Intravitreal Autologous Bone Marrow CD34+ Cell Therapy for Ischemic and Degenerative Retinal Disorders: Preliminary Phase 1 Clinical Trial Findings. Investig. Opthalmology Vis. Sci..

[145] Search of: NCT02638714—List Results—ClinicalTrials.gov [Internet]. NCT02638714.

[146] Search of: NCT01920867—List Results—ClinicalTrials.gov [Internet]. NCT01920867.

[147] Search of: NCT02016508—List Results—ClinicalTrials.gov [Internet]. NCT02016508.

[148] Search of: NCT01518127—List Results—ClinicalTrials.gov [Internet]. NCT01518127.

[149] Search of: NCT01927315—List Results—ClinicalTrials.gov [Internet]. NCT01927315.

[150] Vilela C.A.P., Messias A., Calado R.T., Siqueira R.C., Silva M.J.L., Covas D.T., Paula J.S. (2021). Retinal function after intravitreal injection of autologous bone marrow-derived mesenchymal stromal cells in advanced glaucoma. Doc. Ophthalmol..

[151] Osei-Bempong C., Figueiredo F., Lako M. (2012). The limbal epithelium of the eye--a review of limbal stem cell biology, disease and treatment. BioEssays.

[152] Azmi S.M., Salih M., Abdelrazeg S., Roslan F.F., Mohamed R., Tan J.J., Shaharuddin B. (2020). Human umbilical cord-mesenchymal stem cells: A promising strategy for corneal epithelial regeneration. Regen. Med..

[153] He G.-H., Zhang W., Ma Y.-X., Yang J., Chen L., Song J., Chen S. (2018). Mesenchymal stem cells-derived exosomes ameliorate blue light stimulation in retinal pigment epithelium cells and retinal laser injury by VEGF-dependent mechanism. Int. J. Ophthalmol..

[154] Li D., Zhang J., Liu Z., Gong Y., Zheng Z. (2021). Human umbilical cord mesenchymal stem cell-derived exosomal miR-27b attenuates subretinal fibrosis via suppressing epithelial–mesenchymal transition by targeting HOXC6. Stem Cell Res. Ther..

[155] Search of: NCT03237442—List Results—ClinicalTrials.gov [Internet]. NCT03237442.

